# PFOS mediates immunomodulation in an avian cell line that can be mitigated via a virus infection

**DOI:** 10.1186/s12917-019-1953-2

**Published:** 2019-06-25

**Authors:** Jose M. Castaño-Ortiz, Veerle L. B. Jaspers, Courtney A. Waugh

**Affiliations:** 1 Department of Biology, Norwegian University of Science and Technology (NTNU), Høgskoleringen 5, 7491 Trondheim, Norway; 2 Present address: Catalan Institute for Water Research (ICRA), C/Emili Grahit 101, 17003 Girona, Spain

**Keywords:** PFASs, Immunotoxicity, Birds, PFOS, Gallid herpesvirus-2, Cytokines, qPCR

## Abstract

**Background:**

Per- and polyfluoroalkyl substances (PFASs) are environmentally persistent and bioaccumulative chemicals. Immunomodulation is among the most concerning of toxic effects linked with PFAS exposure in mammalian models. However, no studies had yet shown this to be true in birds. Thus, we designed and conducted the first study to determine if PFASs could cause immunomodulation in birds. Secondly, we wanted to determine the effects on an avian host when exposed not only to immunomodulating chemicals, but also to a viral challenge. The aim, to determine if PFAS mediated immunmodulation functionally affects a pathogen challenge for a host. As innate immune system signalling pathways initiate crucial responses against a pathogen challenge, and are lesser studied than their adaptive counterparts, we focused on these pathways. To provide the first information on this, an in vitro experiment was designed and performed using chicken embryo fibroblasts exposed to perfluorooctane sulfonate (PFOS) (22 ppm) and immune markers characterised before and after being infected with gallid herpesvirus-2 (GaHV-2).

**Results:**

The expression of two pro-inflammatory cytokines, namely interleukin 8 (IL-8) and tumor necrosis factor alpha (TNF-α), the nuclear factor ‘kappa-light-chain-enhancer’ of activated B-cells (NF-κB), and the anti-inflammatory cytokine interleukin 4 (IL-4) were investigated in various scenarios. These results showed that exposure to PFOS decreased immune gene expression in chicken fibroblasts from 36 h post-exposure. Next, it was shown that this decrease could be mitigated by infection with gallid herpesvirus-2, which increased gene expression back to the baseline/control levels.

**Conclusions:**

Not only is this the first study to provide the expected evidence that PFOS has immunomodulatory potential in birds, it also provides unexpected data that virus infections can mitigate this negative effect. Thereby, further research, including in vivo and in situ studies, on the impact of PFOS on host-virus interactions is now warranted, as it has been overlooked and might contribute to our understanding of recent disease outbreaks in wildlife. The mechanisms by which gallid herpesvirus mitigates immunomodulation were beyond the scope of this study, but are now of interest for future study.

**Electronic supplementary material:**

The online version of this article (10.1186/s12917-019-1953-2) contains supplementary material, which is available to authorized users.

## Background

Per- and polyfluoroalkyl substances are highly fluorinated aliphatic chemicals with unique physicochemical properties, which have prompted their use in a wide variety of industrial and commercial applications including surfactants, paints, repellent coatings, fire-fighting foams and ski wax [1–3]. The environmental persistence of PFASs, and their low elimination rates, leads to bioaccumulation in organisms and widespread occurrence across ecosystems [3, 4]. Immunomodulation is one of the most well-known biological effects associated with PFAS exposure in mammals [5]. Yet, there has been limited epidemiological studies to assess a link between PFAS mediated immunomodulation and disease outbreaks in humans [6] and wildlife [7]. Experimental studies, with traditional models, have already demonstrated a range of immune effects, including atrophy of lymphoid organs, and impairment of lymphocyte proliferation or antibody production [8, 9]. The immunomodulation of PFASs in non-mammalian models, such as birds, however, remains understudied, and research to date has focused on adaptive immunity only. For example, Peden-Adams et al. [10] found a decreased response to the phytohemagglutinin (PHA) skin test in white leghorn chickens (*Gallus Gallus domesticus*) following *in ovo* exposure to perfluorooctane sulfonate (PFOS) (1-5 μg/g egg wt). Smits and Nain [11] demonstrated a downregulation of the T-cell dependent antibody response (TDAR) in Japanese quails (*Coturnix japonica*) exposed to perfluorooctanoic acid (PFOA) via drinking water (0.1–10 μg/ml) for eight weeks. However, no relationship was established between PFASs and immunoglobulin levels in plasma of wild white-tailed eagle (*Haliaeetus albicilla*) nestlings from northern Norway [12]. A knowledge gap thereby exists in the understanding of potential effects of PFASs on innate immunity in birds [13].

Innate immunity comprises physical barriers, such as skin and mucosal linings, and a variety of biochemical and cellular responses to infection, including inflammatory responses, and represents the first line of defence against invading pathogens [14]; changes in innate immune function can therefore be hypothesised to impact disease resistance and persistence [15]. Early antiviral responses, for example, are initiated with the activation of pattern recognition receptors (PRRs), by pathogen associated molecular patterns (PAMPs), that eventually trigger the synthesis of various cytokines [14]. For example, gallid herpesvirus-2 (GaHV-2) can interact with toll-like receptor 3 (TLR3) and upregulate the release of interleukin (IL)-8, which is a major pro-inflammatory chemokine [16, 17]. TLR3 ligands induce the activation of NF-κB signalling using the MyD88-independent pathway [18]. It is known from mammalian models that innate signalling cascades can be negatively regulated by PFAS exposure, leading to attenuation of NF-κB activity and associated pro-inflammatory mediators [13]. Research into the mechanistic aspects of immunomodulation has demonstrated that peroxisome proliferator-activated receptor (PPARα) agonists, like PFASs, may interfere with the propagation of infectious signals [19]. Alternatively, PPARα-independent mechanisms may account for PFAS immunomodulation as well [20].

Perfluorooctane sulfonate (PFOS) is the most commonly occurring PFAS in biota, including birds, followed by long-chain perfluoroalkyl carboxylates (PFCAs). In addition, pharmacokinetic properties and immunotoxic effects are the most intensively studied for PFOS, together with perfluorooctanoic acid (PFOA) [21]. Thereby the present study aimed to characterize the effect of PFOS on the antiviral response to infection in birds by using an in vitro model infected with gallid herpesvirus-2 (GaHV-2). GaHV-2, also referred to as Marek’s disease virus (MDV), is an oncogenic α-herpesvirus that causes Marek’s disease, which is a contagious lymphoproliferative disease in domestic fowl [22]. Chickens are primarily exposed to GaHV-2 through inhalation of air-borne viral particles, that then infect antigen-presenting cells, or intermediate cells, from the lung epithelium [23]. GaHV-2 represents a worldwide problem for the poultry industry, and it provides a model of interest for the understanding of virus induced tumorigenesis [22]. Thereby, we designed a targeted study to address the effect of PFOS on the expression of important innate immune signalling components following GaHV-2 infection. Specifically, changes in the relative expression of four immune associated genes were investigated, namely the nuclear factor ‘kappa-light-chain-enhancer’ of activated B-cells (NF-κB), tumor necrosis factor alpha (TNF-α), interleukin 8 (IL-8) and interleukin 4 (IL-4). An avian cell line (chicken (*Gallus gallus*) embryo fibroblasts (CEFs)) were selected as the in vitro model system, due to the global relevance of avian diseases [24] and widespread use of birds in ecotoxicology [25]. In addition, further research, especially in birds, is needed to elucidate potential effects of PFASs on innate immune signalling pathways, and on host-virus interactions [13]. Therefore, we evaluated the expression of NF-κB, TNF-α, IL-8 and IL-4 in CEFs following: (i) infection with GaHV-2, (ii) exposure to PFOS, and (iii) combination of both (exposure to PFOS followed by infection with GaHV-2).

This is of importance because an efficient response to infection in the early recognition of GaHV-2 has been shown to be crucial to the determination of either a positive or negative outcome [26]. Previous studies demonstrated that herpesviruses triggered innate immune signalling cascades when binding to PRRs, including TLRs, retinoic acid-inducible gene I-like receptors (RLRs), and cytosolic DNA sensors [27]. This led to the activation of key transcription factors such as NF-κB, secretion of type I interferons (IFNs) and a set of pro-inflammatory cytokines in chicken tissues and tissue culture cells [27–30]. Any deregulation of these pathways generally led to a more severe outcome in relation to disease severity. We thereby hypothesise that 1) PFOS has the ability to deregulate important innate immune signalling pathways in birds; and 2) that exposure to PFOS can worsen the outcome of an infection in a host.

## Methods

### Cultivation of fibroblasts, GaHV-2, and combined exposure to PFOS and GaHV-2

Chicken embryo fibroblasts (CEFs) were purchased from the American Type Culture Collection (ATCC CRL­12203™ *Gallus gallus* embryo) and were cultivated in Dulbeccos modified Eagle’s s medium (DMEM, Sigma, Oslo, Norway), 5% foetal calf serum (FCS, Sigma, Oslo, Norway), 50 mg/mL Gentamycin (Sigma, Oslo, Norway), 100 units/mL Penicillin and 100 μg/mL Streptomycin. Gallid herpesvirus-2 (GaHV-2) was purchased from LGC Standards GmbH (ATCC® VR-2175™). GaHV-2 was propagated on cultured CEFs in T75 flasks by inoculating the original infected cell seed and incubating for seven days at 39 °C with 5% CO_2_. Only a very mild cytopathic effect was observed after this period. The harvested GaHV-2 stock solutions were kept in cryovials at − 80 °C inside an isopropanol chamber overnight, and then stored in liquid nitrogen for further experimental use.

Cells were seeded at a density of 5.24 × 10^4^ cells/mL in 96-well plates and allowed to rest at 39 °C with 5% CO_2_ for 48 h before exposure. Treatment groups included: (i) infection with GaHV-2; (ii) exposure to perfluorooctane sulfonate (PFOS) (22 ppm-diluted in DMSO 1%, Sigma, St Louis, USA), (iii) a combination of both (i.e. exposure to PFOS (22 ppm) followed by infection with GaHV-2, and (iv) non-exposure control group (media only). PFOS exposure always occurred at 48 h, and GaHV-2 stimulation occurred at 72 h. A ten-fold dilution of the GaHV-2 stock was used as the initial viral titer for well infections (80 μl). Cells were harvested at 96 h post-seed, every 6 h for a 24-h period (Additional file 1: Figure S1) via trypsinization: 2 PBS washes, addition of trypsin-EDTA (50 μl) for 5 min at 39 °C, before resuspension in DMEM media and addition of 100 μl lysis reagent (QIAzol®). Eight biological replicates were arranged per treatment and time point.

### RNA extraction, cDNA conversion and qPCR

RNA was extracted using the miRNeasy Mini Kit (Qiagen, Oslo, Norway). The standard operating procedure from the manufacturer was used. Replicates from the same treatment and time point were pooled into new tubes to obtain sufficient amount of RNA for analysis, resulting in four treatments per time point. The extracted RNA was eluted into RNase-free water and stored overnight at − 20 °C. RNA concentration was determined using a NanoDrop® ND-2000cUV-visible Spectrophotometer (NanoDrop Technologies, Wilmington, USA). Reverse transcription (RT) was performed on a fixed amount of RNA (50 ng/μl) using the miScript II RT kit (Qiagen, Oslo, Norway) and quantitative (q) PCR was conducted using the miScript SYBR green PCR kit (Qiagen, Oslo, Norway). cDNAs were diluted to a final concentration of 1000 pg/μl with nuclease free water and 6 ng of the diluted cDNA was used in each 20 μl qPCR reaction (run in technical duplicates). The mRNA assays included 10 μL SYBR green and 2 μL of each primer (10 μM) (Additional file 2: Table S1) with the following running conditions: 15 min at 95 °C, three step cycling at 15 s at 94 °C, 30 s at 52 °C and 45 s at 68 °C for 50 cycles. After amplification and collection of fluorescence data, melt curve analysis was performed to exclude the possibility of non-specific amplification. Target genes represented a key transcription factor in immune signalling (NF-κB), a classical cytokine marker of inflammation (IL-8), a major pro-inflammatory chemokine (TNF-α), a novel marker of inflammation (TNF-α), and an anti-inflammatory cytokine (IL-4).

### Data analysis

All analyses were performed in R (V 3.3.2) using the specialized package MCMC.qpcr [31]. Raw qPCR data (i.e. Ct values) were represented as molecule counts and described under a Poisson-lognormal error distribution using generalized linear mixed models. This approach is fully flexible for all levels of random and fixed effects, and it enables evaluation of unlimited interactions while increasing the power via simultaneous analysis of all genes in one model. Control genes are not required for this procedure [31] and thus were not implemented in this study. For analysis, a two-way design model was fitted using “treatment” (media only, PFOS only, GaHV-2 only, PFOS/GaHV-2) and “time point” (hours post-infection) as fixed factors (Eq. 1). The model has a single response variable, the natural logarithm of transcript counting rate. The most basic explanatory variable is ‘gene’ which accounts for different levels of expression between genes, the model is then augmented with the gene specific effects of our treatments (“gene:treatment”) and time (“gene:timepoint”), and other random sources of variation (e.g. technical replicates) are also taken into account (“sample”).

1 $$ \mathrm{Ln}\left(\mathrm{rate}\right)\sim \mathrm{gene}+\mathrm{gene}:\mathrm{treatment}+\mathrm{gene}:\mathrm{timepoint}+\mathrm{gene}:\mathrm{timepoint}:\mathrm{treatment}+\mathrm{sample} $$

The Markov Chain Monte Carlo (MCMC) chain was run for 13,000 iterations (the first 3000 were discarded) to estimate the change in target mRNA in response to fixed effects. Results are presented as plots of inferred transcript log_2_abundances (model estimates) arranged by gene, with ± standard deviation (SD) of the estimate. Significant changes in expression were tested with MCMC-based *p*-values for each of the estimated parameters.

## Results

### Effect on basal immune gene expression – PFOS only

**Fig. 1:**
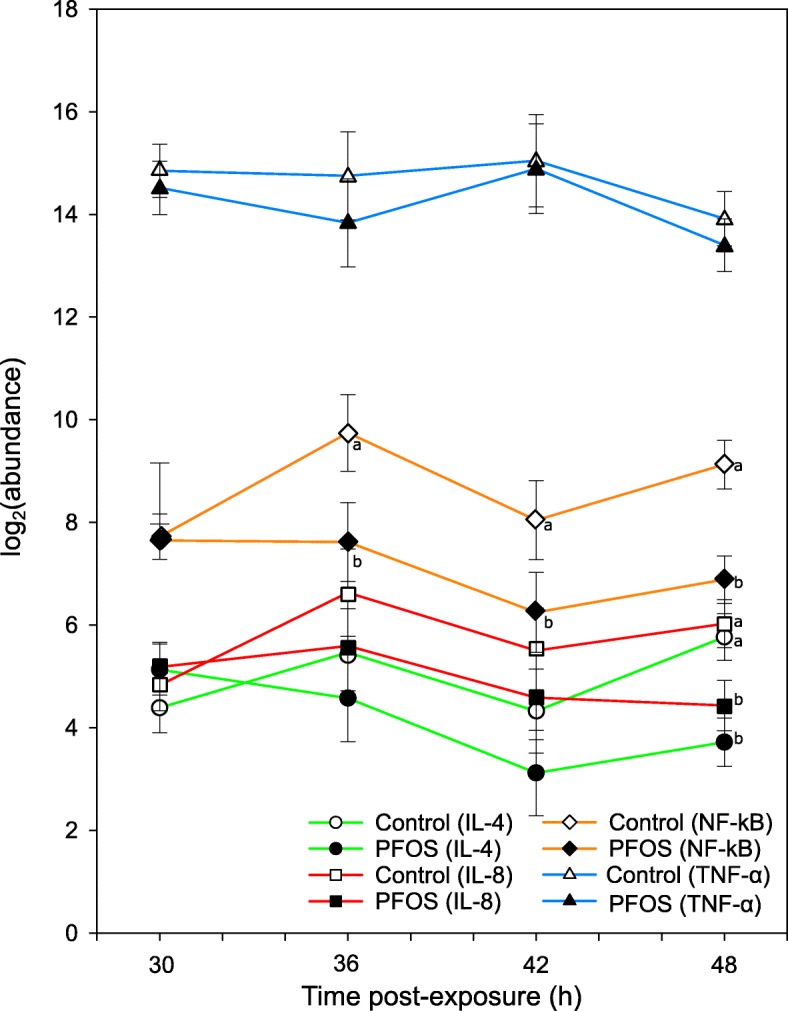
Gene expression profile in response to PFOS only exposure. Overview of log_2_(abundance) of gene transcripts (mRNA expression) in chicken embryo fibroblasts (CEFs) treated with PFOS (    ) versus the non-exposure control (    ). The x-axis denotes duration of the post-exposure period and the y-axis the model estimate for nuclear factor ‘kappa-light-chain-enhancer’ of activated B-cells (NF-κB), interleukin 8 (IL-8), tumor necrosis factor α (TNF-α) and interleukin 4 (IL-4). Model estimates are presented along with its SD in whiskers. Different letters indicate a significant pairwise difference for a given gene

A significant downregulation of NF-kB, IL-8 and IL-4 was found in primary chicken fibroblast cells exposed to PFOS. Following exposure to the pollutant, the basal expression (that is, media only) of the cytokines and transcription factor showed a consistent decrease across all time points (Fig. 1). Differences in relative gene expression were particularly remarkable at 42 h and 48 h post-exposure. Specifically, the basal expression of NF-κB was significantly lower than the control at 36 h and 42 h post-exposure (36 h: *p* < 0.001, 42 h: *p* = 0.007, 48 h: *p* < 0.001). IL-8 expression decreased over time as well, with lower estimates from 36 h and a significant decrease at 48 h post-exposure (*p* = 0.021). A similar pattern applied to IL-4, with lower transcript levels at 48 h (*p* = 0.004). Regarding TNF-α, gene expression estimates in PFOS-exposed cells were similar or lower than in control cells, but no significant downregulation could be inferred.

### Infection of chicken embryo fibroblasts with GaHV-2

**Fig. 2:**
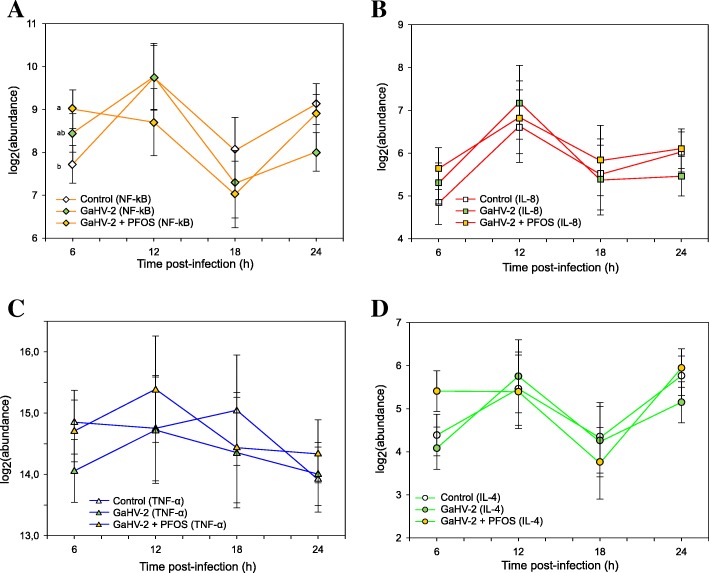
Gene expression profile in response to GaHV-2 only and PFOS/GaHV-2 exposure. log2(abundance) of gene transcripts (mRNA expression) in chicken embryo fibroblasts (CEFs) infected with gallid herpesvirus-2 (GaHV-2) (   ), treated with PFOS/GaHV-2 (   ) versus the non-exposure control (   ). The x-axis denotes duration of the post-exposure period and the y-axis the model estimate for nuclear factor ‘kappa-light-chain-enhancer’ of activated B-cells (NF-κB) (**a**), interleukin 8 (IL-8) (**b**), tumor necrosis factor α (TNF-α) (**c**) and interleukin 4 (IL-4) (**d**). Model estimates are presented along with its standard deviation in whiskers. Different letters indicate a significant pairwise difference for a given gene

The gene expression of the four immune genes (NF- κB, IL-8, TNF-α, IL-4) was determined in chicken embryo fibroblasts: 1) infected with GaHV-2 only and 2) media only control. When these two treatments were compared, they did not yield any statistically significant changes in their expression. This was consistent at all timepoints (i.e. between 6 and 24 h post-infection) (compared to media only; Fig. 2).

### Combined exposure and infection – PFOS/GaHV-2

**Fig. 3:**
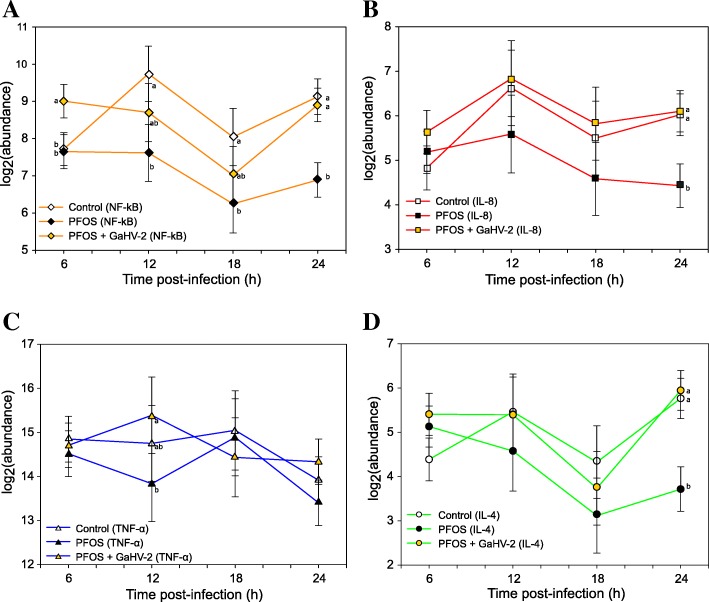
Gene expression profile in response to PFOS only and PFOS/GaHV-2 exposure. log2(abundance) of gene transcripts (mRNA expression) in chicken embryo fibroblasts (CEFs) treated with PFOS (   ), treated with PFOS/GaHV-2 (   ), versus the non-exposure control (   ). The x-axis denotes duration of the post-exposure period and the y-axis the model estimate for nuclear factor ‘kappa-light-chain-enhancer’ of activated B-cells (NF-κB) (**a**), interleukin 8 (IL-8) (**b**), tumor necrosis factor α (TNF-α) (**c**) and interleukin 4 (IL-4) (**d**). Model estimates are presented along with its standard deviation in whiskers. Different letters indicate a significant pairwise difference for a given gene

PFOS only exposed cells (which resulted in a significant decrease in immune gene expression) were next assessed in relation to PFOS/GaHV-2. In this scenario, the PFOS mediated depression of the immune genes was overridden and corrected to normal expression levels when infected with GaHV-2. Specifically, expression significantly increased in PFOS/GaHV-2 cells compared to PFOS only, for NF-κB (*p* = 0.002), IL-8 (*p* = 0.013) and IL-4 (*p* < 0.001) at 24 h post-infection. There was also a trend towards a higher expression of IL-8 after 18 h (*p* = 0.077) (Fig. 3b). For TNF-α, expression levels after 12 h were also significantly higher relative to PFOS only cells (*p* = 0.031). In addition, NF-κB expression at 6 h was upregulated relative to PFOS only (*p* = 0.036) (Fig. 3a).

Exposure to PFOS/GaHV-2, compared to GaHV-2 only, did not yield statistically significant changes in the expression of the majority of the genes, however, there was significantly higher expression of NF-κB at 6 h post-infection (*p* = 0.042) (Fig. 2a).

## Discussion

To investigate the potential of the most dominant PFAS in biota, i.e. PFOS, to modulate the innate immune response and disease resistance, this study utilised an in vitro model using CEFs. Prior to this study, there was no information available on the immunomodulatory properties of PFASs to avian innate immune responses and/or disease.

### PFOS modulation of basal immune gene expression

This study provides the first empirical evidence that PFOS can modulate components of the avian innate immune system. These data shows that the expression of three immune genes in CEFs (i.e. NF-κB, IL-8 and IL-4) were negatively modulated following exposure to PFOS. Changes in expression ranged between 3- and 5-fold decreases by 48 h post-exposure (Fig. 1).

Current knowledge about PFOS-mediated effects on innate immune signalling is limited and mostly based on mammalian models [5]. For example, PFOS attenuated the immune status of exposed mice, with a downregulation of basal hepatic levels of various cytokines, such as TNF-α, IL-4 and IFN-γ [5]. It has been suggested that PPARα ligands, like PFASs, are anti-inflammatory, as receptor binding generally leads to a decrease in the activity of NF-κB and production of linked pro-inflammatory cytokines [13]. In addition, PPARα-independent pathways may partially account for PFOS immunotoxicity as well. For example, PFOS inhibits the degradation of inhibitor of kappa B (I-κB) and the phosphorylation of the NF-κB protein p65 [33]. In line with this, the expression of two pro-inflammatory mediators (i.e. NF-κB and IL-8) were lowered by PFOS exposure in this current study, with TNF-α expression also lower, but not statistically significantly lower (Fig. 1). Regarding IL-4, a T helper cell type 2 (Th2) anti-inflammatory cytokine, previous studies have generally reported increased expression following PFOS exposure in rodents [34] and asthmatic humans [35]. This contrasts with the decrease of IL-4 expression in this current study (Fig. 1d), as well as in a study on murine hepatic macrophages [36]. Altogether, however, these findings suggest that PFOS can modulate the balance between Th1 and Th2 cytokines, towards humoral (Th2-like state) [30] or cellular (Th1-like state) immunity; importantly, either way, this represents a risk factor for disease development [35]. In conclusion, our findings in relation to PFOS exposure in avian cells are in agreement with the reported ability of PFASs to modulate cytokine production in mammalian immune cells [20, 37]. This strengthens our knowledge of the potential of PFOS to interfere with activated immune signalling pathways and indicated a potential pathway of PFOS mediated adversity in avian species.

### Infection of chicken embryo fibroblasts with GaHV-2

In this study, infection with GaHV-2 did not yield detectable changes in immune gene expression between 6 and 24 h post-infection for any of the investigated genes (Fig. 2). This was unexpected based on previous studies using the chicken and GaHV-2 model that reported increased levels of pro-inflammatory mediators, both in vivo [29, 30, 38] and in vitro [32]. These responses probably took place within 24 h post-infection, and it is conceived that antiviral effects are linked to the early synthesis of pro-inflammatory cytokines in CEFs [26, 30]. In vivo studies have shown that as early as 3–4 days post-infection, if not earlier, various innate immune responses are detectable under normal circumstances (i.e. nitric oxide and pro-inflammatory cytokine release in various tissues) [30, 32]. However, in this current study, the inflammatory response following infection may have peaked later than measured (6–24 h), likely due to infection at a lower initial viral titer in this study compared to that in previous studies. Further, during titration of the GaHV-2 only a slight cytopathic effect was noted after 7 days of infection. The exact viral titer could unfortunately not be quantified in this study, neither precisely compared to estimates of viral abundance used in similar infection studies: 100–1000 plaque-forming units (PFU) inoculated in vivo [30, 38] or multiplicity of infection (MOI) = 0.001 in CEF cultures [26]. IOther studies have, however, only detected later responses at the gene expression level (from 48 h) in CEFs upon GaHV-2 infection, though shorter incubation times were not employed in these studies [17, 32]. Therefore, a mismatch between the harvesting time points and the peak response may, at least partially, account for the lack of detectable differences between control and GaHV-2 infected cells regarding pro-inflammatory genes. Higher viral titers and/or longer time points, compared to those used in the current study (Methods 2.1), might thus yield a more detectable response for these endpoints and should be further investigated in the future. In turn for IL-4, an anti-inflammatory cytokine, the fact that pro-inflammatory mediators were not upregulated to a significant extent might account for the lack of expression changes in this study. It has been shown that IL-4 release is negatively regulated during inflammation by pro-inflammatory mediators such as microRNA-155 [39, 40].

### Response of avian cells to combined exposures of PFOS and GaHV-2 infection

While PFOS exposure had a negative effect on IL-8, NF-κB and IL-4 expression (Fig. 3), when the cells were exposed to PFOS/GaHV-2 the response returned to that of an ‘unexposed’ or ‘healthy’ host. This suggests that even though the immune gene expression in this study were below detection in virus exposed cells, the virus triggered a response that actually compensated for the negative effect of PFOS.

A possible explanation is that because the pollutant is known to alter cell membrane properties by increasing its permeability [42], the amount of infected cells in PFOS/GaHV-2 may have increased and increased pro-inflammatory gene expression back up to basal levels (Fig. 3). The upregulation of IL-4 in PFOS/GaHV-2 (Fig. 3d) may be explained by the synergistic production of damage associated molecular patterns (DAMPs), since PFOS exerts cell damage at sublethal concentrations that, in combination with infectious signals, may induce IL-4 as survival factor with antiapoptotic properties [43].

Alternatively, a protective effect of viruses on pollutant toxicity has previously been reported in birds [44]. In that study, mortality in mallard ducklings infected with duck hepatitis virus (DHV) and exposed to pesticides, namely DDT and dieldrin, was lower than in birds exposed to the pesticides only. It was suggested that the compensatory effect could relate to virus induced changes in pollutant metabolism, and this hypothesis was further supported by studies on the interaction of viruses with xenobiotic metabolism [45, 46]. This exact mechanism is however unlikely to apply to this study, since GaHV-2 is not known to increase microsomal enzyme activity [47], PFOS has well-known stability [48] and fibroblasts have limited metabolic activity [49]. Nevertheless, this unexpected interaction between pollutant exposure and virus infections in birds remains an interesting area for further investigation.

Overall, no modulatory effects by PFOS on the response to GaHV-2 could be detected for these endpoints, as both treatments (GaHV-2 only and PFOS/GaHV-2) resembled the media only control (Fig. 2). However, induced inflammatory responses have been shown to be altered by exposure to PFOS in mammalian studies. For example, human leukocytes and mice splenocytes activated with lipopolysaccharide (LPS) had a lower release of TNF-α [20, 41], whereas the inflammatory response to the same endotoxin was enhanced in mice macrophages [36]. These studies suggest that PFASs can affect NF-κB signalling and cytokine release through different mechanisms, as mentioned above [37], but this could unfortunately not be confirmed for GaHV-2 exposed avian cells in the current study, likely due to the mismatch between harvested timepoints and the immune response to infection.

## Conclusions

To our knowledge, this is the first study to address changes in immune gene expression in response to PFOS in any bird host. The current study indicates that PFOS can interfere with key innate immune signalling pathway components in birds (NF-κB, IL-8, TNF-α and IL-4) (Fig. 1). Further, this study has unexpectedly provided evidence that PFOS mediated modulations can be mitigated via a virus infection.

Environmental contaminants have been overlooked as potential causative factors in infectious disease outcomes and severity [50, 51], and future studies should keep the focus on innate functions, since they can impact resistance and they are traded off with other relevant physiological processes [15]. The interesting, seemingly positive (rather than the always assumed negative), interaction between PFOS and GaHV further highlights our lack of knowledge of these processes.

## Additional files

Additional file 1: **Figure S1.** Timeline of pollutant exposure (PFOS) and viral infection with gallid herpesvirus-2 (GaHV-2) in the experiment. Treatment groups are shown in distinctively coloured arrows. In the table below, harvesting timepoints are given as both duration of the exposure period (hours post-exposure) or duration of the viral treatment (hours post-infection). (PDF 87 kb)

Additional file 2: **Table S1.** Primers used for mRNA qPCR analysis. Selected genes included the nuclear factor ‘kappa-light-chain-enhancer’ of activated B-cells (NF-κB1, interleukin 8 (IL-8), tumor necrosis factor alpha (TNF-α) and interleukin 4. Corresponding primer sequences were obtained from Sigma-Aldrich® [52, 53]. (DOCX 13 kb)

## Data Availability

All data generated and analysed during this study are included in the published article (and its Supplementary file 1).

## Abbreviations

cDNA: Complementary DNA
CEFs: Chicken embryo fibroblasts
Ct: Threshold cycle
DAMP: Damage associated molecular pattern
DHV: Duck hepatitis virus
DMSO: Dimethyl sulfoxide
FCS: Fetal calf serum
GaHV-2: Gallid herpesvirus-2
h: Hours
IFN: Inteferon
IL: Interleukin
I-κB: Inhibitor of kappa B
LPS: Lipopolysaccharide
MCMC: Markov chain monte carlo
MDV: Marek’s disease virus
ml: Milliliters
mM: Millimolar
mRNA: Messenger ribonucleic acid
MyD88: Myeloid differentiation primary response 88
NF-kB: Nuclear factor ‘kappa-light-chain-enhancer’ of activated B-cells
ng: Nanograms
PAMP: Pathogen associated molecular pattern
PBS: Phosphate-buffered saline
PCR: Polymerase chain reaction
PFASs: Per- and polyfluoroalkyl substances
PFCAs: Perfluoroalkyl carboxylates
PFOA: Perfluorooctanoic acid
PFOS: Perfluorooctane sulfonate
PPARα: Peroxisome proliferator-activated receptor α
PRR: Pattern recognition receptor
qPCR: Quantitative polymerase chain reaction
RLR: Retinoic acid-inducible gene I-like receptor
RNA: Ribonucleic acid
RT: Reverse transcription
Th1: Helper T cells type 1
Th2: Helper T cells type 2
TLR: Toll-like receptor
TNF: Tumor necrosis factor
