# Supplementary figures and images for: PFOS mediates immunomodulation in an avian cell line that can be mitigated via a virus infection
Source: BMC Vet Res. 2019 Jun 25;15:214. doi: 10.1186/s12917-019-1953-2 (PMC6593586; doi:10.1186/s12917-019-1953-2)

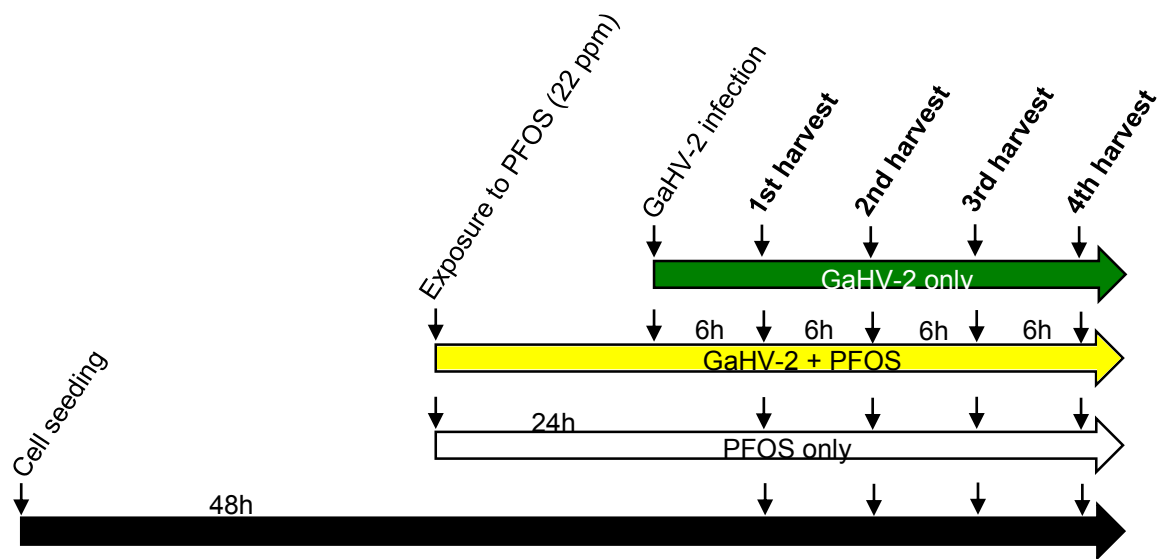

|                         |    |    |    |    |
|-------------------------|----|----|----|----|
| Time post-exposure (h)  | 30 | 36 | 42 | 48 |
| Time post-infection (h) | 6  | 12 | 18 | 24 |

Supplement: Supplementary file 1 — Figure S1. Timeline of pollutant exposure (PFOS) and viral infection with gallid herpesvirus-2 (GaHV-2) in the experiment. Treatment groups are shown in distinctively coloured arrows. In the table below, harvesting timepoints are given as both duration of the exposure period (hours post-exposure) or duration of the viral treatment (hours post-infection). (PDF 87 kb) [file 12917_2019_1953_MOESM1_ESM.pdf]
