# Supplementary material for: PFOS mediates immunomodulation in an avian cell line that can be mitigated via a virus infection
Source: BMC Vet Res. 2019 Jun 25;15:214. doi: 10.1186/s12917-019-1953-2 (PMC6593586; doi:10.1186/s12917-019-1953-2)
Supplement: Supplementary file 2 — Table S1. Primers used for mRNA qPCR analysis. Selected genes included the nuclear factor ‘kappa-light-chain-enhancer’ of activated B-cells (NF-κB1, interleukin 8 (IL-8), tumor necrosis factor alpha (TNF-α) and interleukin 4. Corresponding primer sequences were obtained from Sigma-Aldrich® [52, 53]. (DOCX 13 kb) [file 12917_2019_1953_MOESM2_ESM.docx]

| **Additional file 2: Table S1. Primers used for mRNA qPCR analysis**. Selected genes included the nuclear factor 'kappa-light-chain-enhancer' of activated B-cells (NF-κB1, interleukin 8 (IL-8), tumor necrosis factor alpha (TNF-α) and interleukin 4. Corresponding primer sequences were obtained from Sigma-Aldrich^®^. | | |
| --- | --- | --- |
| Gene | Primer sequence | Reference |
| NF-κB1 | GCAACTATGTTGGACCTGCAAA (Fwd) TGGTGGAAGAAGGTACGTAGG (Rev) | (52) |
| IL-8 | CTGGCCCTCCTCCTGGTT (Fwd)  GCAGCTCATTCCCCATCTTTAC (Rev) | (52) |
| TNF-α | CCCCTACCCTGTCCCACAA (Fwd)  TGAGTACTGCGGAGGGTTCAT (Rev) | (52) |
| IL-4 | GAGAGGTTTCCTGCGTCAAG (Fwd)  TGGTGGAAGAAGGTACGTAGG (Rev) | (53) |
